# Detecting somatic point mutations in cancer genome sequencing data: a comparison of mutation callers

**DOI:** 10.1186/gm495

**Published:** 2013-10-11

**Authors:** Qingguo Wang, Peilin Jia, Fei Li, Haiquan Chen, Hongbin Ji, Donald Hucks, Kimberly Brown Dahlman, William Pao, Zhongming Zhao

**Affiliations:** 1 Department of Biomedical Informatics, Vanderbilt University School of Medicine, Nashville, TN, USA; 2 Center for Quantitative Sciences, Vanderbilt University Medical Center, Nashville, TN, USA; 3 State Key Laboratory of Cell Biology, Institute of Biochemistry and Cell Biology, Shanghai Institutes for Biological Sciences, Chinese Academy of Sciences, Shanghai, China; 4 Department of Thoracic Surgery, Fudan University Shanghai Cancer Center, Shanghai, China; 5 Department of Oncology, Shanghai Medical College, Shanghai, China; 6 Vanderbilt-Ingram Cancer Center, Vanderbilt University Medical Center, Nashville, TN, USA; 7 Department of Cancer Biology, Vanderbilt University School of Medicine, Nashville, TN, USA; 8 Department of Medicine/Division of Hematology-Oncology, Vanderbilt University School of Medicine, Nashville, TN, USA; 9 Department of Psychiatry, Vanderbilt University School of Medicine, Nashville, TN, USA

## Abstract

**Background:**

Driven by high throughput next generation sequencing technologies and the pressing need to decipher cancer genomes, computational approaches for detecting somatic single nucleotide variants (sSNVs) have undergone dramatic improvements during the past 2 years. The recently developed tools typically compare a tumor sample directly with a matched normal sample at each variant locus in order to increase the accuracy of sSNV calling. These programs also address the detection of sSNVs at low allele frequencies, allowing for the study of tumor heterogeneity, cancer subclones, and mutation evolution in cancer development.

**Methods:**

We used whole genome sequencing (Illumina Genome Analyzer IIx platform) of a melanoma sample and matched blood, whole exome sequencing (Illumina HiSeq 2000 platform) of 18 lung tumor-normal pairs and seven lung cancer cell lines to evaluate six tools for sSNV detection: EBCall, JointSNVMix, MuTect, SomaticSniper, Strelka, and VarScan 2, with a focus on MuTect and VarScan 2, two widely used publicly available software tools. Default/suggested parameters were used to run these tools. The missense sSNVs detected in these samples were validated through PCR and direct sequencing of genomic DNA from the samples. We also simulated 10 tumor-normal pairs to explore the ability of these programs to detect low allelic-frequency sSNVs.

**Results:**

Out of the 237 sSNVs successfully validated in our cancer samples, VarScan 2 and MuTect detected the most of any tools (that is, 204 and 192, respectively). MuTect identified 11 more low-coverage validated sSNVs than VarScan 2, but missed 11 more sSNVs with alternate alleles in normal samples than VarScan 2. When examining the false calls of each tool using 169 invalidated sSNVs, we observed >63% false calls detected in the lung cancer cell lines had alternate alleles in normal samples. Additionally, from our simulation data, VarScan 2 identified more sSNVs than other tools, while MuTect characterized most low allelic-fraction sSNVs.

**Conclusions:**

Our study explored the typical false-positive and false-negative detections that arise from the use of sSNV-calling tools. Our results suggest that despite recent progress, these tools have significant room for improvement, especially in the discrimination of low coverage/allelic-frequency sSNVs and sSNVs with alternate alleles in normal samples.

## Background

Rapid advances in next generation sequencing (NGS) technologies, together with the development of powerful computational tools, have transformed biological and biomedical research over the past several years. The transformation has been most apparent in cancer, where the complex landscapes of somatic variants have been investigated in a wide variety of tumor types [1-3]. Most significantly, a number of clinically actionable mutations have been identified as important therapeutic targets in anti-cancer treatments, narrowing the gap between basic research and clinical application. Examples include single nucleotide variants (SNVs) involving codons V600 and L597 in the gene *BRAF* in melanomas, which are associated with sensitivity to *BRAF* and *MEK* inhibitors, respectively [4,5].

A comprehensive knowledge of somatic variants in cancer is indispensable for us to understand tumorigenesis and develop personalized therapies for patients. However, although advances in next-generation sequencing and computational algorithms have led to higher accuracy in somatic SNV (sSNV) calling, some true sSNVs are still difficult to distinguish due to low allele frequencies, artifacts, tumor contamination, inadequate sequencing coverage of genomic regions with high GC content, sequencing errors, and ambiguities in short read mapping, just to name a few. Another confounding factor is clonal heterogeneity that causes variants to be non-uniformly present in tumors [6,7]. Specifically, this difficulty involves two aspects: false-negative sSNVs (true sSNVs not called by the tools) and false-positive sSNVs (spurious sSNVs, either germline SNVs or non-variants).

Somatic SNVs are identified by comparing a tumor sample with a matched normal sample (usually blood from the same patient). Originally, algorithms for identifying sSNVs involved calling variants in the two samples separately, for example, SNVMix [8]. To meet the challenges of sSNV calling, a number of tools with enhanced accuracy have been developed that compare a tumor–normal pair directly at each locus of a possible sSNV, for example, JointSNVMix [9], SomaticSniper 1.0 [10], Strelka [11], and VarScan 2 [12]. In comparison with previous methods, the new tools can effectively differentiate sSNVs from germline events, which vastly outnumber sSNVs and thereby constitute the majority of false calls. Other programs, such as MuTect [13] and EBCall [14], specifically focus on detecting low-allelic-frequency sSNVs that are often missed by existing tools.

Although each of the new tools has been compared with some earlier applications, their relative merits in real applications are largely unknown to investigators, due to incomplete (and sometimes biased) experimental evaluation. For example, the accuracy of MuTect was not benchmarked against VarScan 2, a widely used somatic variant-calling tool released a year earlier. To provide a comparative analysis of sSNV-calling tools, several review articles emerged lately [15,16]. However, these reviews either lacked validation experiments [16] or used only synthetic data [15], and hence are not adequate to guide cancer genome sequencing studies. Further evaluation of these tools’ capability is still urgently required.

Here, we compare the abilities of the recently released tools (Table 1), particularly MuTect and VarScan 2, to detect sSNVs from NGS. We provide an in-depth discussion of the pros and cons of each tool using validated NGS data, so that readers are aware of the kinds of false-positive and false-negative results that may arise. Furthermore, we use simulation data to analyze the ability of each tool to detect sSNVs at different allele frequencies.

**Table 1:** Tools for detecting somatic single nucleotide variants (sSNVs) from next generation sequencing (NGS) data

**Tool**	**Version**	**URL**	**Remark**	**Date**^**a**^	**Ref.**
EBCall	2	https://github.com/friend1ws/EBCall	Uses an empirical Bayesian model to call sSNVs	Mar. 2013	[14]
JointSNVMix	0.8(b2)	http://compbio.bccrc.ca	Joint analysis of tumor/normal pairs	Jan. 2012	[9]
MuTect	1.1.4	http://www.broadinstitute.org/cancer/cga/mutect	Sensitive detection of low allelic-fraction sSNVs	Feb. 2013	[13]
SomaticSniper	1.0.2	http://genome.wustl.edu/software/somaticsniper	High computational efficiency	Dec. 2011	[10]
Strelka	0.4.10.2	ftp://strelka@ftp.illumina.com/	Clean outputs through stringent filtering	May 2012	[11]
VarScan 2	2.3.5	http://varscan.sourceforge.net/	Sensitive detection of high-quality sSNVs	Feb. 2012	[12]

^a^Date: online/electronic publication date.

## Methods

### NGS data

We used whole genome sequencing (WGS) of a melanoma sample and matched blood, whole exome sequencing (WES) of 18 lung tumor-normal pairs and seven lung cancer cell lines to evaluate SNV-calling tools.

The paired-end sequencing of the melanoma sample (90% tumor content) and matched blood was performed on an Illumina Genome Analyzer IIx platform as described [5]. Average coverage was 55.7× and 47.8× for the tumor and matched blood, respectively. From this sample, our previous study identified 339,057 sSNVs using SAMtools [17] pileup. As it is too expensive and labor intensive to validate all those sSNVs, we selected those of functional importance, that is, non-synonymous and stopgain sSNVs, for experimental validation. For each selected sSNV, PCR and direct sequencing was performed using genomic DNA from the same tumor and matched blood samples. The resulting sequences were then analyzed using Mutation Surveyor DNA Variant Analysis Software [18] in addition to manual inspection of the sequence traces. The PCR primers used for direct sequencing of sSNVs are publicly available through the original work [5].

The whole exome data of the 18 lung tumors (approximately 70% to 80% tumor content) and matched normal samples were captured using Agilent SureSelect 38 M kit and sequenced on an Illumina HiSeq 2000 platform as published [19]. On average, 48 Mb paired-end reads were garnered per sample with an average sequencing depth of 63× in target regions. Overall, the sequence reads covered ≥98.9% bases of the target regions by at least one read and ≥79.9% bases by a depth of at least 20×. SAMtools was used to characterize sSNVs in these samples; Sanger sequencing was utilized to validate functionally important sSNVs. We have made the original sequence data available at [20].

We also included NGS data from seven lung cancer cell lines. Two of them, PC-9/S2 and PC-9/BRc1, were sequenced on an Illumina HiSeq 2000 platform using Nimblegen SeqCap Ez Exome Library kit v2 [21]. We obtained 8.4 × 10^9^ bases of short reads (74 bp paired-end) for PC-9/S2 with an average of 232.6× coverage, and 7.8 × 10^9^ bases of short reads for PC-9/BRc1 (216.7× coverage) [21]. The other five cell lines, HCC827, HCC827/R1, HCC827/R2, HCC4006, and HCC4006/ER, were sequenced on an Illumina HiSeq 2000 platform using the Agilent SureSelect 38 Mb kit for whole-exome sequencing. Their average coverage is all >109×. For these seven cell lines, the sequence reads covered ≥98.9% bases of the target regions by at least one read and ≥85.5% bases by a depth of at least 20×. Eight pairs of cell lines were compared to identify sSNVs that were unique to drug sensitivity or drug resistance cell lines (see Additional file 1: Table S2). Specifically, the 'somatic model’ was executed by designating the targeted cell line as 'tumor’ and the cell line to be compared as 'normal’. The sSNVs that resulted from the analysis were then experimentally validated by Sanger resequencing. Cell line DNAs were used as template for PCR amplification. M13-tagged gene-specific primers were designed using Primer 3 software [22]. Sequence chromatograms were analyzed using Mutation Surveyor software [18] and manual inspection. The details can be found in the original work [21].

We also simulated WES of 10 tumor-normal pairs using the profile-based Illumina pair-end Read Simulator (pIRS) [23]. Our simulation procedure and corresponding command lines were described in detail in Additional file 2. We fixed the insert size of the simulated reads at 200 bp. The read length and average coverage were set to 75 bp and 100×, respectively. Additionally, we let the frequency of sSNVs in each sample be 10 times higher than that of indels and structural variants be 10 times less than indels. Because tumor samples carry driver mutations, we let the frequency of SNVs in the tumor be higher than that in the normal sample.

### Alignment

We utilized BWA [24] to align short sequencing reads to the UCSC human reference genome hg19. The default arguments of BWA were applied to the alignment. After the alignment, we ran the software SAMtools [17] to convert the alignment files to a sorted, indexed binary alignment map (BAM) format. Then, we used Picard [25] to mark duplicate reads. To obtain the best call set possible, we also followed the best practice with the software GATK [26] to do realignment and recalibration. The recalibrated alignment files were then used for sSNV detection.

### SNV calling

JointSNVMix [9] uses a command 'train’ to learn the parameters of its probabilistic model. We let the argument *skip_size* of 'train’ be 100 for WES samples and 1,000 for WGS samples to balance its accuracy and computational efficiency. The command 'classify’ in JointSNVMix computes the posterior probability of joint genotypes. In our experiments, we used a non-default argument *post_process*, which was provided in the new version of JointSNVMix, to run 'classify’ to improve its filtering accuracy [27]. The resulting sSNVs with *P(somatic)* >0.999 and *post_process_p_somatic* >0.6 are regarded as high confidence sSNVs. The detailed command lines for the installation and execution of JointSNVMix, as well as other sSNV-detecting tools, are provided in Additional file 3.

MuTect [13], Strelka [11], and SomaticSniper [10] were run in their default settings. dbSNP version 132 and COSMIC v54 were provided to MuTect as its inputs. The sSNVs that were accepted by MuTect were then used as its high confidence (HC) predictions. To obtain SomaticSniper’s HC sSNVs, the outputs of SomaticSniper underwent a filtering procedure as suggested by the tool developers. The recommended configuration was also used to run VarScan 2 [12]. The high confidence outputs of VarScan 2 were applied directly to our analysis.

## Results and discussion

We started with the melanoma tumor sample and its matched normal sample (blood from the same individual) in order to examine the accuracy of the tools in Table 1. We then expanded this effort to a large population of lung tumors and lung cancer cell lines. For these samples, we restricted our discussion to validated sSNVs, which include: (1) true-positive sSNVs: sSNVs predicted by a tool and validated; (2) false-positive sSNVs: sSNVs predicted but not validated; (3) false-negative sSNVs: sSNVs not predicted but validated; and, (4) true-negative sSNVs: sSNVs not predicted and not validated.

### Detecting sSNVs in a melanoma sample

In our previous report on the melanoma sample, 339,057 sSNVs were detected; 1,130 were high-quality non-synonymous/stop gain sSNVs (Phred quality >37, depth >21) [5]. In total, 128 functionally important sSNVs were validated, out of which 119 were true-positive sSNVs and nine were false-positives. This sample harbors the aforementioned driver mutation *BRAF* L597. We ran the six tools (EBCall, JointSNVMix, MuTect, SomaticSniper, Strelka, and Varscan 2) on both the melanoma and matched blood samples. With the exception of EBCall, all these tools successfully rediscovered the *BRAF* L597 mutation.

Table 2 summarizes the results of analyses using these tools. Because they (except EBCall) detected a similar amount of sSNVs from the data, to simplify our assessment, we directly compared each tool’s number of true-positive predictions. As shown in Table 2, VarScan 2 had the highest true-positive rate, missing only one sSNV (at chr19:40886706) in its high confidence (HC) setting. This missed sSNV was detected by VarScan 2 initially. It was filtered out later by VarScan 2 due to a significant amount of mismatches flanking the mutated site. Aside from VarScan 2, other tools did not report this specific sSNV either.

**Table 2:** Number of validated true/false sSNVs detected using the five tools in a melanoma sample

**Tool**	**All sSNVs**		**High confidence sSNVs**	
	**# TP**	**# FP**	**# TP**	**# FP**
JointSNVMix	119	2	108	0
MuTect	119	9	115	0
SomaticSniper	116	4	104	0
Strelka	117	0	113	0
Varscan 2	119	6	118	2

FP: false-positive sSNVs; TP: true-positive sSNVs.

MuTect had the second best performance, missing four real sSNVs (Table 2). The reasons that MuTect rejected these sSNVs were various, including 'nearby gap events’ and 'alternate allele in normal’, among others. For the sSNV rejected for 'alternate allele in normal’, only one out of 42 (2.4%) reads was actually altered at this site in the blood sample, indicating the stringent filtering strategy of MuTect. At this site in the tumor, 21 (28%) out of 75 reads support this somatic event (9 on the forward strand and 12 on the reverse), exhibiting strong evidence for its existence. In addition to MuTect, JointSNVMix and SomaticSniper also missed this sSNV, while VarScan 2, together with Strelka, correctly reported it (see Additional file 1: Table S1).

The alternate allele for a somatic SNV is observed in the normal sample typically as a result of sample contamination, for example, circulating tumor cells in blood, normal tissue contaminated with adjacent tumor. Sequencing error and misalignment can also contribute false mutation-supporting reads to the normal. Because sample contamination is hard to prevent during sample preparation step, it is important for an sSNV-calling tool to tolerate to some extent the presence of low level mutation allele in normal sample in order not to miss authentic sSNVs. Hence, while using a tool less tolerant to alternate allele in the normal, for example, MuTect, researchers are advised to check the sSNVs rejected for alternate allele in the normal, especially when characterizing sSNVs from low purity samples.

Table 2 also shows that VarScan 2 reported two false-positive sSNVs (at chr6:128294851 and chr19:7141752, respectively). Both sSNVs exhibited stand bias, that is, their mutated bases are present in only one allele. Due to the importance of strand bias, we leave the in-depth discussion of this topic to the next section.

It may be worth mentioning that EBCall, as shown in Table 1, uses a set of normal samples to estimate sequencing errors with which to infer the discrepancy between the observed allele frequencies and expected errors. Although this design might improve sSNV calling [14], a potential problem is that unmatched error distribution between normal references and target samples can adversely affect variant calling. If investigators do not have normal references with the same/similar error rate as the target tumors, this strategy inevitably fails. This may explain our experimental observations, in which EBCall (in its default setting) failed to identify the majority of sSNVs despite the fact that the normal references we used were sequenced from the same Illumina platform as the tumors. Due to its lower-than-expected accuracy, we therefore excluded EBCall from Table 2, and, hereafter, we did not include EBCall in our comparison.

### Identifying sSNVs in lung tumors and lung cancer cell lines

Next, we evaluated the five tools using WES data of 18 lung tumor-normal pairs [19] and seven lung cancer cell lines [21]. For these 43 WES samples, 118 putative sSNVs were validated as true-positives. The majority of these sSNVs had decent coverage in both tumor and normal samples, while 26 of them (all from the 18 lung tumors) were covered by <8 reads in the normal samples and were therefore designated as 'low quality’ in Table 3. Of note, here we used the default read-depth cutoff of VarScan 2, that is, eight in the normal samples, to denote an sSNV as either high or low quality.

**Table 3:** Number of validated true/false sSNVs detected using the five tools in the lung cancer samples

**Tool**	**Lung tumors (*n*** **= 18)**			**Lung cancer cell lines (*n*** **= 7)**			**Total**^**a**^		
	**TP**		**FP**	**TP**		**FP**	**TP**		**FP**
	**# High quality**	**# Low quality**		**# High quality**	**# Low quality**		**# High quality**	**# Low quality**	
JointSNVMix	30	17	6	49	0	11	79	17	17
MuTect	30	19	11	47	0	7	77	19	18
SomaticSniper	33	17	10	47	0	9	80	17	19
Strelka	27	17	8	51	0	10	78	17	18
Varscan 2	35	8	5	51	0	13	86	8	18

^a^Total is the sum of #sSNVs found in lung tumors and lung cancer cell lines.

FP: false-positive sSNVs; TP: true-positive sSNVs.

For these WES samples, 64% (59 out of 92) high-quality validated sSNVs were reported by all the five tools, less than the 82% of the sSNVs that they shared on the melanoma sample. Among the five tools, VarScan 2 identified the most high-quality sSNVs (Table 3). For characterization of low quality ones, however, VarScan 2 was inferior to the other tools primarily due to its stringent read-depth cutoffs and our application of its high-confidence setting in this study. MuTect detected the most low-quality sSNVs, but at a cost of an elevated false positive rate, as indicated in column 3 of Table 3. For the sSNVs missed by MuTect but identified by VarScan 2, 10 out of 14 (7 from the lung tumors, another 7 from the lung cancer cell lines) had support reads in the normal samples. This result confirmed our previous observation that MuTect appeared to be more conservative than VarScan 2 in reporting sSNVs with alternate alleles in the normal samples.

For these 43 WES samples, 160 putative sSNVs were false-positives. The large number of false-positive sSNVs of these data allowed us to examine the typical false calls of these tools. Table 3 shows that overall these tools had similar false detection rates. Additionally, as a result of a preference to detect more sSNVs in higher coverage data, Varscan 2 called 13 false-positive sSNVs in the seven lung cancer cell lines, more than MuTect and other tools. Varscan 2’s tendency to call more sSNVs in higher quality data was also manifested on the 18 lung tumors, where it also characterized more high quality sSNVs than other tools. Nine (69%) out of the 13 false calls by Varscan 2 from the seven cell lines have alternate alleles in the normal samples. Similarly, the majority (>63%) of false-positive sSNVs detected by the other four tools from the seven cell lines have support reads in the normal (see Additional file 1: Table S2), indicating that the challenge to discriminate sSNVs with alternate alleles in normal samples remains to be illuminated.

As demonstrated in the section above, when calling sSNVs, another potential source of false-positives is strand bias. Here, we specifically call an sSNV whose alternate alleles all come from one strand a strand-biased sSNV. The phenomenon of stand bias is common with Illumina sequencing data. For example, among the nine false sSNVs validated for the melanoma sample, six exhibited strand bias. The discrimination of strand-biased sSNVs from artifacts is another current challenge. Some tools, for example, Strelka, discard strand-biased sSNVs, especially those of low quality, so that investigators do not waste resources on validating potential wild-type mutations. Another strategy used in many tools, for example, VarScan 2 and MuTect, is to keep them for users to decide whether to keep or discard. MuTect implemented a strand-bias filter to stratify reads by direction and then detect SNVs in the two datasets separately. This filter allows MuTect to reject spurious sSNVs with unbalanced strands effectively. From our lung cancer and melanoma samples, MuTect identified four strand-biased sSNVs in total, VarScan 2 reported five, and none was found by Strelka. The number of false-positive sSNVs among these detections was 1 and 2 for MuTect and VarScan 2, respectively. For the two aforementioned false-positives identified by VarScan 2 in the melanoma sample, the reads supporting the reference allele were highly biased to the forward strand (one SNV: 22+/3-; another: 21+/2-), while the reads supporting the alternate allele were all biased to the reverse (one: 0+/11-; another: 0+/10-) , hence indicating a sign of duplicity. MuTect successfully filtered out both false-positives.

As shown in Table 3, from the 18 lung tumors, MuTect reported a total of 11 false-positive sSNVs, the most among the five tools. Among these false-positive detections, two were not reported by other tools, and were thus unique to MuTect (see Additional file 4: Figure S1). One of these two MuTect-specific sSNVs exhibited strand bias in addition to a low coverage in the normal sample, while the other had low coverage in both tumor and normal samples.

### Detecting sSNVs at different allele frequencies

Due to cost, researchers often choose only a small subset of high-quality and functionally important sSNVs for experimental validation. As a result, publicly available validation results of low allelic-frequency sSNVs are rare. With the lack of experimental data, here, we used simulation data instead to evaluate these tools’ capabilities to identify sSNVs at different allele fractions.

We simulated 10 pairs of whole exome sequencing samples at coverage of 100× (see Additional file 2). Then, we ran the tools (listed in Table 1) to identify sSNVs from these data. Because few sSNVs within the captured regions were at low allele fractions (approximately 1% with allele frequency <0.15), we utilized all high quality sSNVs, both inside and outside the target regions, to evaluate these tools’ sensitivity. Here, an sSNV is considered high quality if it has at least two reads supporting the alternate allele in disease sample, ≥20 base quality (Phred), and a minimum 8× coverage.

**Figure 1:**
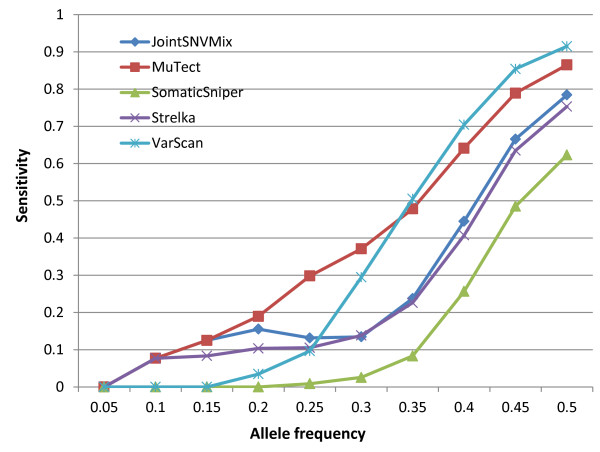
**Sensitivity as a function of mutation allele frequency for five sSNV-detecting tools.** Given an allele frequency value *f*, the sensitivity of a tool *T* (either JointSNVMix, MuTect, SomaticSniper, Strelka, or VarScan 2) is calculated as: *S*_*T*_ *= N*_*T*_*/N*_*f*_ where *N*_*f*_ is the total number of sSNVs with sequencing depth ≥8, the number of alternate allele-supporting reads ≥2 in the disease sample, and an allele frequency less than *f*, and *N*_*T*_ is the number of sSNVs that the tool *T* identified out of these *N*_*f*_ point mutations.

Figure 1 shows the sensitivity of these tools as a function of sSNV allele frequencies. Given an allele frequency value *f*, the sensitivity of a tool *T* (that is, JointSNVMix, MuTect, SomaticSniper, Strelka, or VarScan 2), is calculated as: *S*_*T*_ *= N*_*T*_*/N*_*f*_, where *N*_*f*_ is the total number of sSNVs with a frequency less than *f*, depth ≥8 and the number of alternate allele-supporting reads ≥2 in the disease sample; *N*_*T*_ is the number of sSNVs that the tool *T* identifies out of these *N*_*f*_ point mutations. From Figure 1, we can see that MuTect detected more sSNVs at <0.34 frequencies than the other tools. For sSNVs at higher allele fractions, VarScan 2 outperformed MuTect and other tools in its sensitivity ranking, which is consistent with our previous observation involving real tumor samples where VarScan 2 was the most sensitive software for detecting high-quality sSNVs.

In order to interrogate ultra-rare sSNVs, for example, point mutations with frequencies <1/100 or even as low as 1/10,000 alleles, investigators are advised to utilize targeted deep sequencing [28-30] instead of WES or WGS, where the average coverage is relatively low. However, targeted deep sequencing and related tools are beyond the scope of this paper, as our focus here is on tools designed primarily for WGS and WES, which are currently the most popular technologies for investigating sSNVs as well as other genetic variations in cancer.

## Conclusions

The accurate characterization of sSNVs in tumor-normal matched samples is critical to cancer research and personalized cancer therapy. In this paper, we have evaluated the capability of new sSNV detection tools. Our discussion focused on MuTect and VarScan 2 in particular due to their relatively high accuracy [13,15] and widespread application to NGS-based cancer studies. Of note, our analysis of their performance on real tumor samples was restricted to a relatively small dataset, which included 237 successfully validated sSNVs and 169 false-positive ones.

Our results highlighted the distinct performance of these sSNV-detecting tools. Although a large number of sSNV calls, especially high-quality ones, were shared among these tools, the overall observation across our three types of benchmark data demonstrated that VarScan 2 excelled at the detection of high-quality sSNVs (high coverage and allele frequency), while MuTect outperformed all other tools in detecting low-quality ones. Their distinct features hence suggest that a combination of multiple tools, for example, MuTect with VarScan 2, may benefit real projects by identifying more sSNVs.

Herein, we also provided an in-depth discussion of the types of sSNVs that a tool might have missed and the typical false-positive detections by these tools. Our evaluation using real tumor sequencing data demonstrated that in comparison with VarScan 2, MuTect missed more sSNVs with alternate allele in normal samples. Moreover, both MuTect and VarScan 2 were flawed in discerning sSNVs with alternate allele in normal sample and sSNVs exhibiting strand bias; therefore, we suggest investigators select such sSNVs with caution for follow-up experimental validation.

We have also examined these sSNV detection tools at different allele frequencies using simulation data. Our results showed that MuTect outperformed other tools in characterizing low allelic-fraction sSNVs. However, existing tools, including MuTect, all missed the majority of sSNVs at low allele frequencies on our simulation data. Thus, to interrogate cancer genomes in exquisite detail, there is still significant room for improvement.

## Abbreviations

HC: High confidence; NGS: Next generation sequencing; SNV: Single nucleotide variant; sSNV: Somatic SNV; WES: Whole exome sequencing; WGS: Whole genome sequencing.

## Competing interests

The authors declare that they have no competing interests.

## Authors’ contributions

QW and ZZ conceived and designed the project. QW and PJ carried out the data analysis. HJ, HC, FL, KD, DH, and WP contributed experimental data. QW, PJ, WP, and ZZ drafted the manuscript. All authors read and approved the final manuscript.

## Supplementary Material

Additional file 1: Tables S1-S3 The somatic single nucleotide variants (sSNVs) identified using the tools JointSNVMix, MuTect, SomaticSniper, Strelka, and VarScan 2 from the melanoma sample were provided in **Table S1.** The sSNVs identified in the seven lung cancer cell lines were provided in **Table S2.** The number of sSNVs detected in the simulation data was provided in **Table S3.** Click here for file

Additional file 2 Simulation of whole exome sequencing. Click here for file

Additional file 3 Detailed command lines for installing and running sSNV-detecting tools. Click here for file

Additional file 4: Figure S1 The number of false-positive sSNVs identified in 18 lung tumors. In total, 94 sSNVs were validated as false-positives for this data. Another program, VarScan 2, reported five out of 94 false positive sSNVs, all or most of which were also reported by these four tools and, hence, were not depicted here so as to make the figure readable. Click here for file
