# Supplementary material for: Detecting somatic point mutations in cancer genome sequencing data: a comparison of mutation callers
Source: Genome Med. 2013 Oct 11;5(10):91. doi: 10.1186/gm495 (PMC3971343; doi:10.1186/gm495)
Supplement: Additional file 2 — Simulation of whole exome sequencing. [file gm495-S2.docx]

**Additional file 2**

**Detecting somatic point mutations in cancer genome sequencing data: a comparison of mutation callers**

Qingguo Wang^1^, Peilin Jia^1,2^, Fei Li^3^, Haiquan Chen^4,5^, Hongbin Ji^3^, Donald Hucks^6^, Kimberly Brown Dahlman^6,7^, William Pao^6,8§^, Zhongming Zhao^1,2,7,9§^

^1^Department of Biomedical Informatics, Vanderbilt University School of Medicine, Nashville, TN, USA

^2^Center for Quantitative Sciences, Vanderbilt University Medical Center, Nashville, TN, USA

^3^State Key Laboratory of Cell Biology, Institute of Biochemistry and Cell Biology, Shanghai Institutes for Biological Sciences, Chinese Academy of Sciences, Shanghai, China

^4^Department of Thoracic Surgery, Fudan University Shanghai Cancer Center, Shanghai, China

^5^Department of Oncology, Shanghai Medical College, Shanghai, China

^6^Vanderbilt-Ingram Cancer Center, Vanderbilt University Medical Center, Nashville, TN, USA

^7^Department of Cancer Biology, Vanderbilt University School of Medicine, Nashville, TN, USA

^8^Department of Medicine/Division of Hematology-Oncology, Vanderbilt University School of Medicine, Nashville, TN, USA

^9^Department of Psychiatry, Vanderbilt University School of Medicine, Nashville, TN, USA

^§^ Corresponding author.

Email addresses:

QW: [qingguo.wang@vanderbilt.edu](mailto:qingguo.wang@vanderbilt.edu)

PJ: [peilin.jia@vanderbilt.edu](mailto:peilin.jia@vanderbilt.edu)

FL: lifei120616@gmail.com

HC: [hqchen1@yahoo.com](mailto:hqchen1@yahoo.com)

HJ: [hbji@sibcb.ac.cn](mailto:hbji@sibcb.ac.cn)

DH: [donald.hucks@Vanderbilt.Edu](mailto:donald.hucks@Vanderbilt.Edu)

KD: [kimberly.b.dahlman@Vanderbilt.Edu](mailto:kimberly.b.dahlman@Vanderbilt.Edu)

WP: william.pao@Vanderbilt.Edu

ZZ: [zhongming.zhao@vanderbilt.edu](mailto:zhongming.zhao@vanderbilt.edu)

**Simulation of whole exome sequencing to evaluate tools for detecting somatic point mutations**

(Last updated: 8/23/2013)

1. **Background**

Whole exome sequencing (WES) is the most widely used sequencing technology of today for investigating single nucleotide variants (SNVs) as well as other genetic variations in human cancer. We simulated WES data in order to evaluate tools for calling somatic SNVs. An advantage of simulated data over real data, in which the complete set of somatic events is not available, is that with the knowledge of exact loci of all SNVs, we are able to calculate accurately the sensitivities and false discovery rates of SNV-detecting tools.

1. **Simulation of a single sample**

When we began to collect data for evaluating SNV-calling tools in May 2012, no software was available for WES simulation yet. So we utilized a whole genome sequencing-simulating software, profile based Illumina pair-end Read Simulator (pIRS) [1], to create experimental reads. pIRS simulates reads with empirical base-calling and GC%-depth profiles trained from real Illumina sequencing data in order to make its reads fit the properties of real data. Figure 1 below provides the flow chart of our simulation approach.

**Figure 1 Simulation of a single whole exome sequencing (WES) sample.**

Extract sequence *R* from the BAM file

A WES BAM file

Use pIRS to insert SNVs and indels into the sequence *R*, from which to generate paired-end reads

Convert coordinates of SNVs

FASTQ files and hg19 positions of all variants

As shown in Figure 1, we started with a BAM file, which was created by aligning paired-end WES reads of a human cancer cell line using BWA [2] to human reference genome hg19. From this BAM file, we first used SAMtools [3] mpileup and bcftools in the SAMtools package to extract DNA sequence segments of the cell line, which ideally consist of all targeted exonic regions of the genome. Let *R* denote the extracted DNA sequence segments.

Next, we ran pIRS to insert SNVs, small insertions and deletions (indels), and structural variations into *R* to obtain a new variant-harboring sequence *R’*. Then, using *R’* as reference, we ran pIRS to generate paired-end sequencing reads (in FASTQ format). For each simulated WES sample, we fixed the insert size of the simulated reads at 200bp. The read length and average coverage were set to 75bp and 100×, respectively. Additionally, we let the frequency of SNVs in each sample be 10 times higher than that of indels and structural variants be 10 times less than indels.

Because we used *R’* instead of hg19 as reference sequence to run pIRS, the positions of resulting SNVs, which were assigned by pIRS according to their coordinates in *R’*, have to be converted to their corresponding positions in hg19. Let *x* be the coordinate of a SNV in *R’* and *y* be its corresponding position in hg19. We used the following Formula (1) to calculate *y*,

*y=HASH(x)* (1)

where *HASH* is a hash function, the implement of which includes several script files such as *convert-loci-snp.pl* (see Appendix).

The final outputs of the pipeline in Figure 1 for each simulated WES sample are therefore two FASTQ files and hg19 positions of all variants. The command lines corresponding to the above steps are provided in detail in the Appendix.

One drawback of this simulation approach is that if reads in the initial BAM file are mapped to intronic regions of the human genome, these intronic regions can be incorporated into *R* and hence likely be sampled by pIRS to produce sequencing reads. Another potential caveat is that the sizes of the exon-harboring segments in *R* may differ from the length of the corresponding target regions. However, although this approach is flawed, with bed files supplied by human exon capture kit, the influence of untargeted regions on benchmark studies of SNV-calling tools can be effectively reduced by excluding the variants identified outside the target regions.

1. **Simulation of paired samples**

Figure 1 only depicts the simulation of one WES sample. But somatic SNVs are identified through comparison of a pair of samples (typically a disease sample and its matched normal sample). To simulate a pair of disease-normal matched samples, a two-step procedure should be followed theoretically: (a) using the approach described in Figure 1 to simulate a normal sample; (b) using the BAM file of the created normal sample as input to simulate a matched disease sample. However, because of the variants inserted into the normal sample in Step (a), in Step (b) the Formula (1) above cannot be used to calculate the hg19 positions of the variants in the disease sample anymore. The positions of somatic variants need add or deduct sites/sizes of the variants in the normal samples so as to infer their hg19 coordinates.

To simplify the conversion of variant’s coordinates for the disease sample, we simulated both the disease and normal samples directly from the same BAM file, as illustrated in Figure 2. To differentiate the disease sample from the normal sample, we let the frequency of SNVs in the disease be higher than that in the normal, considering that disease samples usually carry driver mutations. One consequence of this simplification is that this design is not able to insert germline variants into the matched samples. All the germline variants in the simulated sample pairs come from the initial BAM file, from which they were created.

**Figure 2 Simulation of paired WES samples.**

A WES BAM file

(a) Simulated normal sample

(b) Simulated disease sample

1. **Results**

We simulated 10 disease-normal paired samples. The number of somatic SNVs inserted in each disease sample is provided in Table 1 below.

Table 1 Number of somatic SNVs in 10 simulated disease samples

| Sample No. | 1 | 2 | 3 | 4 | 5 | 6 | 7 | 8 | 9 | 10 |
| --- | --- | --- | --- | --- | --- | --- | --- | --- | --- | --- |
| #All SNVs | 3,057 | 3,112 | 3,131 | 3,030 | 3,026 | 3,097 | 3,040 | 2,953 | 3,116 | 3,031 |
| #Callable SNVs^1^ | 1,335 | 1,367 | 1,374 | 1,337 | 1,315 | 1,396 | 1,367 | 1,292 | 1,339 | 1,343 |
| #SNVs in target regions | 259 | 265 | 286 | 260 | 256 | 281 | 281 | 268 | 295 | 275 |

^1^Callable SNVs are ones with ≥6× coverage in disease sample, ≥1 support read, and ≥20 base quality (Phred).

For SNVs with ≥6 read depth and ≥1 support read in the disease sample, and ≥20 Phred base quality, we defined them as callable SNVs. The following text used callable SNVs and SNVs in the target regions to illustrate allele frequencies of the simulated SNVs.

Table 2 and Figure 3 provide the average number/percentage of somatic SNVs per sample at different mutation allele frequencies. As expected, the allele fractions of the majority (>90%) of somatic SNVs were in the range of 0.3 to 0.6. Table 2 also shows that the SNVs at <0.2 allele frequencies were very few: ~14 callable per sample. They were even fewer within the target regions. The most of the low allelic-fraction SNVs, either callable or uncallable, were located in regions where coverage was low, i.e. outside the target regions. Hence, to compare specifically SNV-calling tools for detecting SNVs at <0.2 allele frequencies, readers are advised to simulate data at higher coverage and/or mutation rates.

Table 2 Average number of somatic SNVs per sample at different mutation allele frequencies

| Allele frequency range | # Callable SNVs (%) | # SNVs in the target regions (%) |
| --- | --- | --- |
| [0.0, 0.1) | 3 .0 (0.2) | 1.4 (0.5) |
| [0.1, 0.2) | 10.8 (0.8) | 0.5 (0.2) |
| [0.2, 0.3) | 23.9 (1.8) | 1.0 (0.4) |
| [0.3, 0.4) | 111.8 (8.5) | 11.5 (4.3) |
| [0.4, 0.5) | 560 (42.7) | 119.9 (45.2) |
| [0.5, 0.6) | 554 (42.3) | 119.5 (45.0) |
| [0.6, 0.7) | 68.7 (5.2) | 10.7 (4.0) |
| [0.7, 0.8) | 7.3 (0.6) | 0.6 (0.2) |
| [0.8, 0.9) | 2.3 (0.2) | 0.0 (0.0) |
| [0.9, 1.0) | 2.1 (0.2) | 0.3 (0.1) |

­­

**Figure 3 The percentage of somatic SNVs as a function of mutation allele frequency (averaged on 10 simulated WES samples). The data of the figure is from Table 2.**

These 10 simulated samples have been used in a previous study [4] to compare SNV-calling tools, including JointSNVMix, SAMtools, SomaticSniper 1.0, Strelka and VarScan 2 (the popular tool MuTect was not publicly available yet at the time of our previous study). For sensitivity, false discovery rate, and speed of these tools on this simulation data, interested readers are referred to [4].

**References**

1. Hu X, Yuan J, Shi Y, Lu J, Liu B, Li Z, Chen Y, Mu D, Zhang H, Li N, Yue Z, Bai F, Li H, Fan W: **pIRS: Profile-based Illumina pair-end reads simulator**. *Bioinformatics* 2012, **28**:1533–1535.

2. Li H, Durbin R: **Fast and accurate short read alignment with Burrows-Wheeler transform**. *Bioinformatics* 2009, **25**:1754–1760.

3. Li H, Handsaker B, Wysoker A, Fennell T, Ruan J, Homer N, Marth G, Abecasis G, Durbin R: **The Sequence Alignment/Map format and SAMtools**. *Bioinformatics* 2009, **25**:2078–2079.

4. Wang Q, Zhao Z: **A comparative study of methods for detecting small somatic variants in disease-normal paired next generation sequencing data**. In *2012 IEEE Int Work Genomic Signal Process Stat GENSIPS*; 2012:38–41.

**Appendix Command lines of WES simulation**

############## (1) Extract WES reference sequence #####

## 1.1 Create a Perl script vcfutils-ref.pl by replacing subroutines vcf2fq and v2q_post_process in vcfutils.pl (in the SAMtools package) with the codes below, ##

sub vcf2fq {

my %opts = (d=>3, D=>100000, Q=>10, l=>5);

getopts('d:D:Q:l:', \%opts);

die(qq/

Usage: vcfutils.pl vcf2fq [options] <all-site.vcf>

Options: -d INT minimum depth [$opts{d}]

-D INT maximum depth [$opts{D}]

-Q INT min RMS mapQ [$opts{Q}]

-l INT INDEL filtering window [$opts{l}]

\n/) if (@ARGV == 0 && -t STDIN);

my ($last_chr, $seq, $qual, $last_pos, @gaps);

my $_Q = $opts{Q};

my $_d = $opts{d};

my $_D = $opts{D};

my %het = (AC=>'M', AG=>'R', AT=>'W', CA=>'M', CG=>'S', CT=>'Y',

GA=>'R', GC=>'S', GT=>'K', TA=>'W', TC=>'Y', TG=>'K');

$last_chr = '';

while (<>) {

next if (/^#/);

my @t = split;

if ($last_chr ne $t[0]) {

&v2q_post_process($last_chr, \$seq, \$qual, \@gaps, $opts{l})

if ($last_chr);

($last_chr, $last_pos) = ($t[0], 0);

$seq = $qual = '';

@gaps = ();

}

die("[vcf2fq] unsorted input\n") if ($t[1] - $last_pos < 0);

my ($ref, $alt) = ($t[3], $1);

my ($b, $q);

$q = $1 if ($t[7] =~ /FQ=(-?[\d\.]+)/);

$seq .= $ref;

$qual .= $q;

$last_pos = $t[1];

}

&v2q_post_process($last_chr, \$seq, \$qual, \@gaps, $opts{l});

}

sub v2q_post_process {

my ($chr, $seq, $qual, $gaps, $l) = @_;

for my $g (@$gaps) {

my $beg = $g->[0] > $l? $g->[0] - $l : 0;

my $end = $g->[0] + $g->[1] + $l;

$end = length($$seq) if ($end > length($$seq));

substr($$seq,$beg,$end-$beg)=lc(substr($$seq,$beg,$end-$beg));

}

print ">$chr\n"; &v2q_print_str($seq);

}

## 1.2 Run the following commands ##

samtools mpileup -uf hg19.fa WES.example.bam | bcftools view -cg - > exome.bcf

perl vcfutils-ref.pl vcf2fq exome.bcf > exome-ref.fq

######################### (2) Run pIRS ################

## Simulate normal sample. The input file exome-ref.fq was created by the command above ##

/bin/pIRS_110/bin/pirs diploid -i exome-ref.fq -s 0.000001 -d 0.0000001 -v 0.000000001 -o normal_ref_seq
/bin/pIRS_110/bin/pirs simulate -M 1 -i exome-ref.fq -I normal_ref_seq.snp.indel.inversion.fa.gz -Q 33 -l 75 -x 100 -m 200 -v 10 -o normal_

## Simulate tumor sample ##

/scratch/wangq4/bin/pIRS_110/bin/pirs diploid -i exome-ref.fq -s 0.00001 -d 0.000001 -v 0.00000001 -o tumor_ref_seq
/bin/pIRS_110/bin/pirs simulate -M 1 -i exome-ref.fq -I tumor_ref_seq.snp.indel.inversion.fa.gz -Q 33 -l 75 -x 100 -m 200 -v 10 -o tumor_

############### (3) Convert variant coordinates #########

## 3.1 Create a Perl script vcfutils-ref-pos.pl by replacing subroutine vcf2fq in the vcfutils.pl (in the SAMtools package) with the code below, ##

sub vcf2fq {

my %opts = (d=>3, D=>100000, Q=>10, l=>5);

getopts('d:D:Q:l:', \%opts);

die(qq/

Usage: vcfutils.pl vcf2fq [options] <all-site.vcf>

Options: -d INT minimum depth [$opts{d}]

-D INT maximum depth [$opts{D}]

-Q INT min RMS mapQ [$opts{Q}]

-l INT INDEL filtering window [$opts{l}]

\n/) if (@ARGV == 0 && -t STDIN);

my ($last_chr, $seq, $qual, $last_pos, @gaps);

my $_Q = $opts{Q};

my $_d = $opts{d};

my $_D = $opts{D};

my %het = (AC=>'M', AG=>'R', AT=>'W', CA=>'M', CG=>'S', CT=>'Y',

GA=>'R', GC=>'S', GT=>'K', TA=>'W', TC=>'Y', TG=>'K');

$last_chr = '';

while (<>) {

next if (/^#/);

my @t = split;

if ($last_chr ne $t[0]) {

($last_chr, $last_pos) = ($t[0], 0);

$seq = $qual = '';

@gaps = ();

}

die("[vcf2fq] unsorted input\n") if ($t[1] - $last_pos < 0);

my ($ref, $alt) = ($t[3], $1);

my ($b, $q);

$q = $1 if ($t[7] =~ /FQ=(-?[\d\.]+)/);

$seq .= $ref;

$qual .= $q;

$last_pos = $t[1];

print "$last_chr\t$last_pos\t$ref\n";

}

}

## 3.2 Run the following command to prepare a file for hashing. The input file exome.bcf was created previously ##

perl vcfutils-ref-pos.pl vcf2fq exome.bcf > exome-ref.pos

## 3.3 Create a Perl script convert-SNV-loci.pl as follows ##

#!/usr/bin/perl -w

my $snp_file = shift(@ARGV);

my $exome_pos_file = shift(@ARGV);

my %loci_hash;

open(IN, $snp_file);

while(<IN>){

my @t = split;

next if(!$t[0]);

$loci_hash{$t[0].'_'."$t[1]$t[2]"}=$t[3];

}

close(IN);

$last_chr = '';

$idx = 1;

open(IN, $exome_pos_file);

while(<IN>){

my @t = split;

if ($last_chr ne $t[0]){

($last_chr, $idx) = ($t[0], 1);

}

if (exists($loci_hash{$t[0].'_'."$idx$t[2]"})){

my $var = $loci_hash{$t[0].'_'."$idx$t[2]"};

print "$t[0]\t$t[1]\t$t[1]\t$t[2]\t$var\n";

}

$idx=$idx+length($t[2]);

}

close(IN);

## 3.4 Run the following commands to convert SNV coordinates into hg19 positions through a hash table ##

perl convert-SNV-loci.pl tumor_snp.lst exome-ref.pos > tumor_snp_hg19.lst

perl convert-SNV-loci.pl normal_snp.lst exome-ref.pos > normal_snp_hg19.lst

## The file tumor_snp_hg19.lst and normal_snp_hg19.lst contains correct hg19 positions of all SNVs inserted into the simulated sample pair.
