# Supplementary material for: Detecting somatic point mutations in cancer genome sequencing data: a comparison of mutation callers
Source: Genome Med. 2013 Oct 11;5(10):91. doi: 10.1186/gm495 (PMC3971343; doi:10.1186/gm495)
Supplement: Additional file 3 — Detailed command lines for installing and running sSNV-detecting tools. [file gm495-S3.docx]

**Summary of Commands to Call Somatic SNVs in Tumor/Normal-Paired Samples**

(Last updated: 8/29/2013)

1. **Summary of recently published somatic SNV-calling tools**

| **Tool** | **Version** | **URL** | **SNV** | **Indel** | **CNV** | **Ref.** |
| --- | --- | --- | --- | --- | --- | --- |
| EBCall | 2 | <https://github.com/friend1ws/EBCall> | √ | √ |  | [1] |
| JointSNVMix | 0.8(b2) | <http://compbio.bccrc.ca> | √ |  |  | [2] |
| MuTect | 1.1.4 | <http://www.broadinstitute.org/cancer/cga/mutect> | √ |  |  | [3] |
| SomaticSniper | 1.0.2 | <http://genome.wustl.edu/software/somaticsniper> | √ |  |  | [4] |
| Strelka | 0.4.10.2 | <ftp://strelka@ftp.illumina.com/> | √ | √ |  | [5] |
| VarScan | 2.3.5 | <http://varscan.sourceforge.net/> | √ | √ | √ | [6] |

SNV: single nucleotide variant. Indel: insertion and deletion. CNV: copy number variation.

1. **EBCall**

**2.1 Installation**

wget <https://github.com/friend1ws/EBCall/archive/master.zip>

unzip EBCall-master.zip

cd EBCall-master

vi config.sh ///change the path to SAMtools and R

make

chmod +x ebCall_v2.sh

**2.2 Variant calling**

///The following command line is from URL: <https://github.com/friend1ws/EBCall>

./ebCall_v2.sh <full path to tumor.bam> <full path to normal.bam> <full path to output directory> <full path to a file listing of a set of normal (BAM file)>

1. **JointSNVMix**

**3.1 Installation**

Download JointSNVMix (<http://code.google.com/p/joint-snv-mix/downloads/detail?name=JointSNVMix-0.8-b2.tar.gz&can=2&q>=)

tar xzvf JointSNVMix-0.8-b2.tar.gz

setpkgs -a python ///Set the path to python. ‘setpkgs' is a special command provided in ACCRE (Vanderbilt Advanced Computing Center for Research & Education).

python setup.py build

python setup.py install --home=/mylocaldir/JointSNVMix-0.8-b2

cp jsm.py jsm2.py

vi jsm2.py ///add these two lines: import sys

///sys.path.append('/mylocaldir/JointSNVMix-0.8-b2/lib/python')

**3.2 Variant calling**

setpkgs –a python

python jsm2.py train hg19.fa normal.bam tumor.bam JointSNVMix.cfg --model=snvmix2 --skip_size=100

///Suggest to use skip_size=1000 for whole genome sequencing (WGS).

python jsm2.py classify hg19.fa normal.bam tumor.bam --parameters_file=JointSNVMix.cfg --out_file=JointSNVMix.output --post_process --somatic_threshold=0.2

**3.3 Filtering**

awk '{if(NR>1 && $10+$11>0.999 && $18>0.6)print}' JointSNVMix.output > JointSNVMix.output.hc

1. **MuTect**

**4.1 Installation**

Download MuTect, dbSNP version 132 and COSMIC version 54 from Broad Institute: <http://www.broadinstitute.org/cancer/cga/mutect_download>

**4.2 Variant calling**

///Set Java1.6 environment

setpkgs -a java_1.6

///Run MuTect

java -Xmx20g -jar muTect-1.1.4.jar --num_threads 8 --analysis_type MuTect -R hg19.fa -cosmic chr.hg19_cosmic_v54_120711.vcf -dbsnp chr.dbsnp_132_b37.leftAligned.vcf --input_file:normal nomal.bam --input_file:tumor tumor.bam --out cal_stats.out --vcf mutation.vcf -cov coverage.wig.txt --enable_extended_output

///For WES data, provide genomic intervals to MuTect

java -Xmx20g -jar muTect-1.1.4.jar --num_threads 8 --analysis_type MuTect -R hg19.fa -cosmic chr.hg19_cosmic_v54_120711.vcf -dbsnp chr.dbsnp_132_b37.leftAligned.vcf --input_file:normal nomal.bam --input_file:tumor tumor.bam --out cal_stats.out --vcf mutation.vcf -cov coverage.wig.txt --enable_extended_output --intervals exome_target_regions.bed

1. **SomaticSniper**

**5.1 Installation**

///Install somatic-sniper

git clone --recursive git://github.com/genome/somatic-sniper.git

cd somatic-sniper

export SAMTOOLS_ROOT=/mylocaldir/samtools-0.1.6

cmake .

make

///Install bam-readcount

git clone --recursive git://github.com/genome/bam-readcount.git

export SAMTOOLS_ROOT=/mylocaldir/samtools-0.1.17

cmake .

make

**5.2 Variant calling**

/mylocaldir/bam-somaticsniper -q 1 -Q 15 -F classic -f hg19.fa tumor.bam normal.bam snp.classic

**5.3 Filtering**

///The following filtering steps were suggested by tool developers (<https://sites.google.com/site/kchengenomics/software/somaticsniper>).

/mylocaldir/samtools-0.1.6/samtools pileup -sivf tumor.bam > indel.pileup

perl /mylocaldir/somatic-sniper/src/scripts/snpfilter.pl --snp-file snp.classic --indel-file indel.pileup --out-file snp.SNPfilter

perl /mylocaldir/somatic-sniper/src/scripts/prepare_for_readcount.pl --snp-file snp.SNPfilter

/mylocaldir/bam-readcount/bin/bam-readcount -b 15 -f hg19.fa -l snp.SNPfilter.pos tumor.bam > snp.SNPfilter.pos.rc

perl /mylocaldir/somatic-sniper/src/scripts/fpfilter.pl --snp-file snp.SNPfilter --readcount-file snp.SNPfilter.pos.rc

perl /mylocaldir/somatic-sniper/src/scripts/highconfidence.pl --snp-file snp.SNPfilter.fp_pass

1. **Strelka**

**6.1 Installation**

wget <ftp://strelka@ftp.illumina.com/v0.4.10.2/strelka_workflow-v0.4.10.2.tar.gz>

tar xzvf strelka_workflow-v0.4.10.2.tar.gz

mv strelka_workflow-v0.4.10.2 strelka_workflow

cd strelka_workflow

make

///Create two configuration files, strelka_config_bwa_WGS.ini (isSkipDepthFilters = 0) and strelka_config_bwa_WES.ini (isSkipDepthFilters = 1) for WGS and WES data, respectively – suppose you use BWA for alignment.

**6.2 Variant calling**

/mylocaldir/strelka_workflow/configureStrelkaWorkflow.pl --tumor=tumor.bam --normal=normal.bam --ref=hg19.fa --config=/mylocaldir/strelka_workflow/strelka/etc/strelka_config_bwa_targeted.ini

cd strelkaAnalysis

make -j 8 ///Using 8 cores on the local machine

///Alternatively, use command: make –j 8 –C <full path to strelkaAnalysis>

1. **VarScan**

**7.1 Installation**

wget <http://sourceforge.net/projects/varscan/files/VarScan.v2.3.5.jar/download>

**7.2 Variant calling**

samtools mpileup -C50 -Bf hg19.fa nomal.bam > normal.bam.mpileup

samtools mpileup -C50 -Bf hg19.fa tumor.bam > tumor.bam.mpileup

java -jar VarScan.v2.3.5.jar somatic normal.bam.mpileup tumor.bam.mpileup --output-snp snp --output-indel indel

**7.3 Filtering**

java -jar VarScan.v2.3.5.jar somaticFilter snp --indel-file indel --output-file snp.filtered

java -jar VarScan.v2.3.5.jar processSomatic snp.filtered

java -jar VarScan.v2.3.5.jar processSomatic indel

**References**

1. Shiraishi Y, Sato Y, Chiba K, Okuno Y, Nagata Y, Yoshida K, Shiba N, Hayashi Y, Kume H, Homma Y, Sanada M, Ogawa S, Miyano S: **An empirical Bayesian framework for somatic mutation detection from cancer genome sequencing data**. *Nucleic Acids Res* 2013.

2. Roth A, Ding J, Morin R, Crisan A, Ha G, Giuliany R, Bashashati A, Hirst M, Turashvili G, Oloumi A, Marra MA, Aparicio S, Shah SP: **JointSNVMix: a probabilistic model for accurate detection of somatic mutations in normal/tumour paired next-generation sequencing data**. *Bioinformatics* 2012, **28**:907–913.

3. Cibulskis K, Lawrence MS, Carter SL, Sivachenko A, Jaffe D, Sougnez C, Gabriel S, Meyerson M, Lander ES, Getz G: **Sensitive detection of somatic point mutations in impure and heterogeneous cancer samples**. *Nat Biotechnol* 2013, **31**:213–219.

4. Larson DE, Harris CC, Chen K, Koboldt DC, Abbott TE, Dooling DJ, Ley TJ, Mardis ER, Wilson RK, Ding L: **SomaticSniper: identification of somatic point mutations in whole genome sequencing data**. *Bioinformatics* 2012, **28**:311–317.

5. Saunders CT, Wong W, Swamy S, Becq J, Murray LJ, Cheetham RK: **Strelka: Accurate somatic small-variant calling from sequenced tumor-normal sample pairs.** *Bioinformatics* 2012.

6. Koboldt DC, Zhang Q, Larson DE, Shen D, McLellan MD, Lin L, Miller CA, Mardis ER, Ding L, Wilson RK: **VarScan 2: somatic mutation and copy number alteration discovery in cancer by exome sequencing**. *Genome Res* 2012, **22**:568–576.
