# Supplementary figures and images for: Detecting somatic point mutations in cancer genome sequencing data: a comparison of mutation callers
Source: Genome Med. 2013 Oct 11;5(10):91. doi: 10.1186/gm495 (PMC3971343; doi:10.1186/gm495)

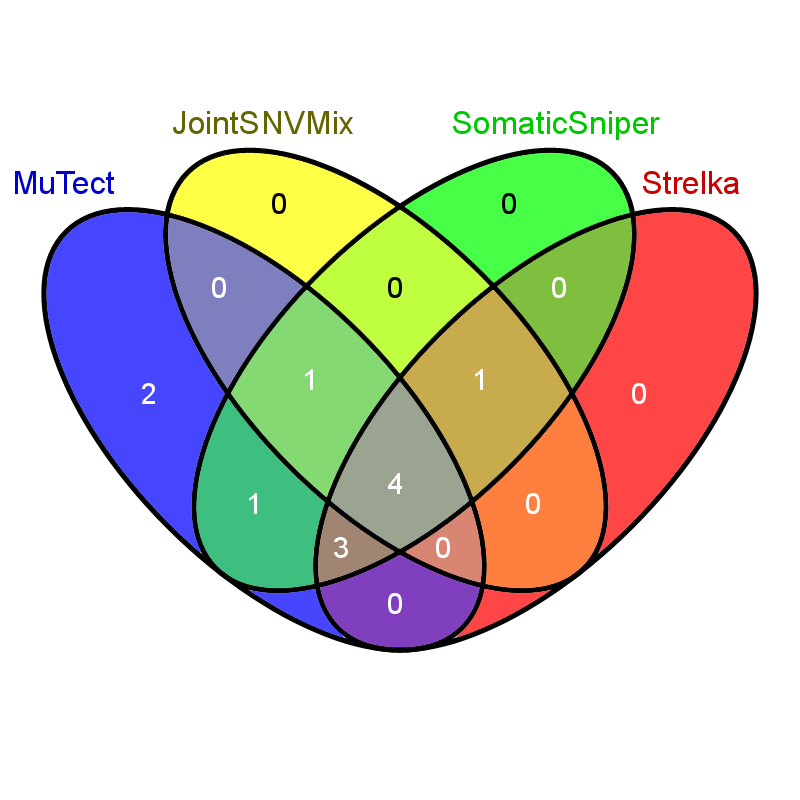

Supplement: Additional file 4: Figure S1 — The number of false-positive sSNVs identified in 18 lung tumors. In total, 94 sSNVs were validated as false-positives for this data. Another program, VarScan 2, reported five out of 94 false positive sSNVs, all or most of which were also reported by these four tools and, hence, were not depicted here so as to make the figure readable. [file gm495-S4.png]
